# Multi-Walled Carbon Nanotubes Improved Development during In Vitro Multiplication of Sugarcane (*Saccharum* spp.) in a Semi-Automated Bioreactor

**DOI:** 10.3390/plants10102015

**Published:** 2021-09-26

**Authors:** Monserrat Sorcia-Morales, Fernando Carlos Gómez-Merino, Lino Sánchez-Segura, José Luis Spinoso-Castillo, Jericó Jabín Bello-Bello

**Affiliations:** 1 Colegio de Postgraduados Campus Córdoba, Carretera Federal Córdoba-Veracruz km. 348, Veracruz 94946, Mexico; monserrat.sorciam@gmail.com (M.S.-M.); fernandg@colpos.mx (F.C.G.-M.); jlspinoso@gmail.com (J.L.S.-C.); 2 CINVESTAV, Departamento de Ingeniería Genética, Unidad Irapuato, Libramiento Norte Carretera, Irapuato-León km. 9.6, Guanajuato 36824, Mexico; lino.sanchez@cinvestav.mx; 3 CONACYT—Colegio de Postgraduados Campus Córdoba, Carretera Federal Córdoba-Veracruz km. 348, Veracruz 94946, Mexico

**Keywords:** in vitro culture, carbon content, chlorophyll, macro- and micronutrients, hormesis

## Abstract

Carbon nanotubes play an important role in plant biotechnology due to their effects on the growth and differentiation of cells, tissues, organs, and whole plants. This study aimed to evaluate the effect of multi-walled carbon nanotubes (MWCNTs) during in vitro multiplication of sugarcane (*Saccharum* spp.) using a temporary immersion system. Morphological characterization of MWCNTs was carried out under a transmission electron microscope. Different concentrations (0, 50, 100, 200 mg L^−1^) of MWCNTs were added to Murashige and Skoog liquid culture medium in the multiplication stage. At 30 d of culture, number of shoots per explant, shoot length, number of leaves per shoot, total chlorophyll, dry matter percentage, carbon percentage, and macro- and micronutrient content were evaluated. Results showed an increase in the development of sugarcane shoots at concentrations of 100 and 200 mg L^−1^ MWCNT. Total chlorophyll content increased at concentrations of 50 and 100 mg L^−1^ MWCNT, whereas macro- and micronutrient content was variable at the different MWCNT concentrations. Results suggest a hormetic effect, characterized by stimulation at low concentrations. In conclusion, the use of low concentrations of MWCNTs had positive effects on development, total chlorophyll, carbon percentage, and macro- and micronutrient (N, Ca, S, Fe, Cu, Zn and Na) contents during in vitro multiplication of sugarcane and may have a potential use in other species of agricultural interest.

## 1. Introduction

Nanotechnology in agriculture is important for the development of nanopesticides, nanofertilizers, nanogrowth regulators, and nanomaterials to improve agricultural production [1]. Nanomaterials are characterized by an ordered assembly of their atoms to form structures on a manometer scale of between 1 to 100 nm [2,3]. Some examples of carbon nanostructures include fullerene, graphene, nanoparticles, nanofibers, and nanotubes [4,5]. Carbon nanotubes (CNTs) are rolled up sheets of graphene forming a hollow cylindrical structure, and they are classified into single-walled carbon nanotubes (SWCNTs) and multi-walled carbon nanotubes (MWCNTs) [6,7]. Compared to SWCNTs, MWCNTs have higher density, tensile strength, and electrical conductivity [8], as well as lower toxicity in plant cells [9]. Some effects of CNTs on plants have been described, notably their positive effect on development, nutritional status, and photosynthesis. However, the effects of CNTs on plants depend on the species, CNT type, concentration, developmental stage and culture conditions [10].

On the other hand, the effects of MWCNTs on plants have only been evaluated in hydroponic cultures of broccoli (*Brassica oleracea* L.), sunflower (*Helianthus annuus* L.), and marijuana (*Cannabis sativa* L.) [11,12]. An alternative to more accurately study the effects of CNTs on plants is by in vitro plant tissue culture. This technique allows the manipulation of cells, tissues, organs, or whole plants under aseptic and controlled conditions. Some recent studies have reported on the administration of MWCNTs in in vitro cultures of sorghum (*Sorghum bicolor* L. Moench), switchgrass (*Panicum virgatum* L.), thyme (*Thymus daenensis*), and African lily (*Agapanthus praecox*) [13,14,15]. However, the physiological and biochemical effects and mechanisms of CNTs in plants have not been fully elucidated. This study aimed to evaluate the effect of carbon nanotubes (CNTs) during in vitro multiplication of sugarcane (*Saccharum* spp.) cv. Mex 69-290 using a temporary immersion system.

## 2. Results

### 2.1. Characteristics of Carbon Nanotubes

**Figure 1:**
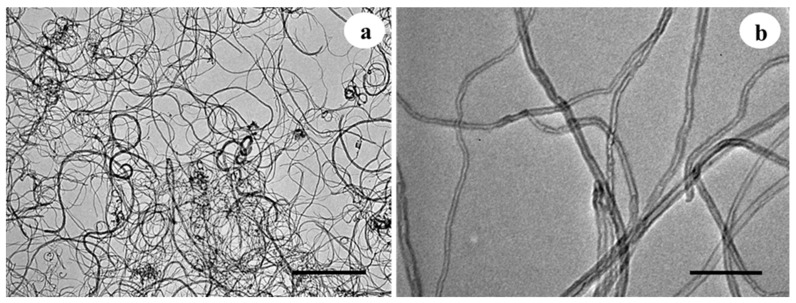
Multi-walled carbon nanotubes (MWCNTs) observed by transmission electron microscopy (TEM) using different magnifications. (**a**) Black bar = 1000 nm. (**b**) Black bar = 100 nm.

The MWCNTs formulation is powder based and with a carbon purity of 98%. Transmission Electron Microscopy (TEM) characterization corroborates the manufacturing dimensions with an outer diameter between 6–13 nm, average length between 2.5–20 μm, and preparation by chemical vapor deposition (Figure 1).

### 2.2. Effect of MWCNTs on Shoot Development, and Chlorophyll and Carbon Content

**Figure 2:**
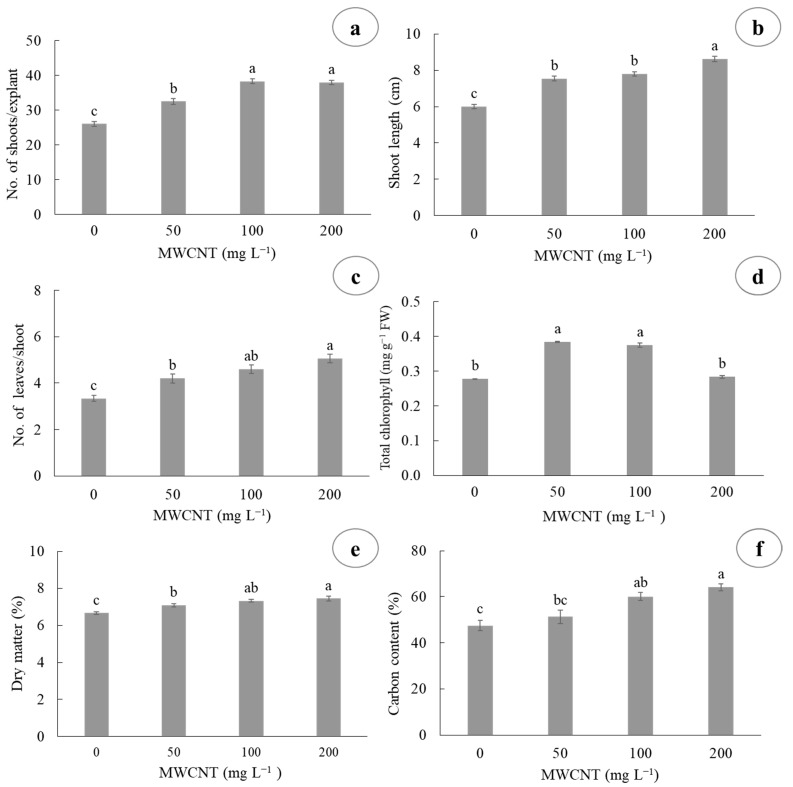
Effect of multi-walled carbon nanotubes (MWCNTs) on shoot development and total chlorophyll and carbon content of sugarcane (*Saccharum* spp.) cv. Mex 69-290 cultured in vitro in temporary immersion. (**a**) Shoots per explant, (**b**) shoot length, (**c**) leaves per shoot, (**d**) total chlorophyll, (**e**) dry matter, and (**f**) carbon content at 30 days of culture. Results are shown as mean ± SE (standard error). Means with a different letter are significantly different (Tukey, *p* ≤ 0.05).

**Figure 3:**
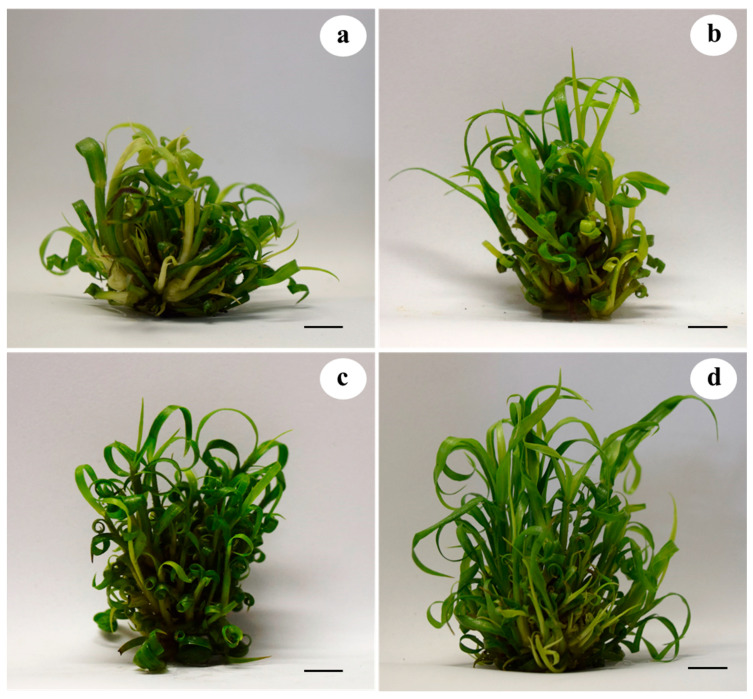
Effect of different MWCNT concentrations on in vitro shoot development of sugarcane (*Saccharum* spp.) cv. Mex 69-290 at 30 days in temporary immersion; (**a**–**d**) 0, 50, 100, and 200 mg L^−1^ MWCNT, respectively. Black bar = 1 cm.

The administration of different MWCNT concentrations had a contrasting effect on the number of shoots per explant, shoot length, number of leaves per shoot, total chlorophyll (Chl) content, dry matter (DM) percentage, and carbon (C) percentage in sugarcane shoots cultured in temporary immersion bioreactors (Figure 2). The highest number of shoots per explant was obtained at the concentrations of 100 and 200 mg L^−1^ MWCNT, with 38.33 and 37.93 shoots per explant, respectively, whereas the lowest number of shoots was observed in the control treatment, with 26.06 shoots per explant (Figure 2a). Regarding shoot length, the highest shoot height was observed at the concentration of 200 mg L^−1^ MWCNT, with 8.61 cm in length, whereas the shortest length was observed in the control treatment, with 6.01 cm in length (Figure 2b). The highest number of leaves per shoot was observed with the concentrations of 100 and 200 mg L^−1^ MWCNT, with 4.6 and 5.06, respectively, whereas the lowest number of leaves was obtained in the control treatment, with 3.33 leaves per shoot (Figure 2c). For total Chl content, the highest amount was observed at the concentrations of 50 and 100 mg L^−1^ MWCNT, with 0.38 and 0.37 mg g^−1^ FW, respectively, whereas the lowest total Chl content was observed at the concentrations of 0 and 200 mg L^−1^ MWCNT, with 0.27 and 0.28 mg g^−1^ FW, respectively (Figure 2d). For DM percentage, the highest content was observed at the concentrations of 100 and 200 mg L^−1^ MWCNT, with 7.31 and 7.44% DM, respectively, whereas the lowest content was obtained in the control treatment, with 6.65% DM (Figure 2e). For C percentage, the highest values were obtained with the concentrations of 100 and 200 mg L^−1^ MWCNT, with 60.00 and 64.07%, respectively, whereas the lowest C content was obtained with the concentrations of 0 and 50 mg L^−1^ MWCNT, with 47.39 and 51.25% carbon, respectively (Figure 2f). In addition, the administration of different MWCNT concentrations had an effect on multiplication rate in sugarcane shoots cultured in temporary immersion bioreactors (TIBs) (Figure 3).

### 2.3. Effect of Carbon Nanotubes on Macro- and Micronutrient Content

A significant effect of MWCNTs was observed on the content of macronutrients N, Ca, and S, as well as on the content of micronutrients Fe, Cu, Zn, and Na (Table 1). The highest N contents were observed at concentrations of 0, 50, and 100 mg L^−1^ MWCNT, with 47,900, 45,800, and 42,400 mg kg^−1^, whereas the lowest content was found at concentration of 200 mg L^−1^, with 39,500 mg kg^−1^. For the Ca element, the highest content was obtained with the 200 mg L^−1^ treatment followed by the treatment at 100 mg L^−1^ MWCNT, with 1848 and 1627 mg kg^−1^, respectively, whereas the lowest Ca contents were at 0 and 50 mg L^−1^ MWCNT, with 1425.42 and 1561.81 mg kg^−1^, respectively. For the macronutrient S, the highest contents were obtained at concentrations of 100 and 200 mg L^−1^ MWCNT, with values of 3267.52 and 3487.07 mg kg^−1^, respectively, whereas the lowest S content was obtained in the treatment without MWCNT, with 3032.37 mg kg^−1^. The macronutrients P and Mg did not show any difference among the treatments. Regarding micronutrients, the highest Fe contents were obtained with the 0, 50, and 100 mg L^−1^ MWCNT treatments, with 299.49, 318.00, and 322.49 mg kg^−1^, whereas the lowest Fe content was found in the treatment with 200 mg L^−1^ MWCNT, with 281.86 mg kg^−1^. For the micronutrient Cu, the highest content was obtained in the control treatment, with 6.41 mg kg^−1^, whereas the lowest Cu content was found in the shoots treated with 100 mg L^−1^ MWCNT, with values between 3.22–3.39 mg kg^−1^.

For the element Zn, the highest contents were in the 50 and 100 mg L^−1^ MWCNT treatments, with 74.45 and 76.65 mg kg^−1^, respectively, whereas the lowest contents were at 0 and 200 mg L^−1^ MWCNT, with 69.73 and 68.98 mg kg^−1^, respectively. The micronutrients Mn, B, and Ni did not show any difference among the treatments. Finally, the highest Na contents were obtained in shoots treated with 0 and 50 mg L^−1^ MWCNT, with 508.29 and 515.24 mg kg^−1^, respectively, whereas the lowest contents were obtained with the concentrations of 100 and 200 mg L^−1^ MWCNT, with 322.45 and 352.95 mg kg^−1^, respectively.

## 3. Discussion

### 3.1. Plant Development and Carbon Content

This study shows the effect of MWCNTs during in vitro multiplication of sugarcane. The increase in multiplication rate, shoot length, and number of leaves at concentrations of 100 and 200 mg L^−1^ MWCNT could be due to increased uptake of nutrients and organic compounds such as sucrose, growth regulators, and vitamins, among others. In this regard, Khodakovskaya et al. [16], working with tobacco (*Nicotina tabacum*) callus in vitro, observed an increase in biomass of between 55–64% at a concentration range of 5–500 μg mL^−1^ MWCNT. Pandey et al. [13], in sorghum (*Sorghum bicolor* L. Moench) and switchgrass (*Panicum virgatum*) seeds in vitro, were able to increase the germination rate and root and shoot length at a concentration of 200 μg mL^−1^ MWCNT. Similarly, Seddighinia et al. [17], in bitter melon (*Momordica charantia*), obtained greater development of shoot and root length, as well as fresh and dry biomass content during germination at 200 mg L^−1^ MWCNT. This suggests an internalization of the carbon nanotubes in the tissues that also contribute to the increase in % DW. The authors of [18] reported that, in hopbush (*Dodonaea viscosa* L.), MWCNTs increased the percentage of seed germination as well as fresh and dry weight of roots and stems at concentrations between 10 and 200 mg L^−1^ MWCNT. Another study [14] on thyme (*Thymus daenensis* celak) in vitro, found an increase in the fresh and dry weight of the stems while their height was three times greater than that of the control in seedlings developed with 250 μg mL^−1^ of MWCNT; however, concentrations of 500–2000 μg mL^−1^ MWCNT had a negative effect on their development.

In this study, the increase in dry weight occurred in response to a higher MWCNT concentration, probably due to a greater accumulation of organic and inorganic compounds that are transported in the tissues by MWCNTs. The major source of carbon was supplied in the form of sucrose and other organic compounds in the MS culture medium (glycine, myo-inositol, nicotinic acid, pyridoxine, and thiamine), the growth regulators Kinetin (KIN), Indoleacetic acid (IAA), and Benzylaminopurine (BAP) and by the C content of the MWCNT treatments. The increase in the percentage of total C is related to the increase in MWCNT concentration.

### 3.2. Chlorophyll Content

The increase in total Chl content at concentrations of 50 and 100 mg L^−1^ MWCNT could be related to higher photosynthetic activity. In this regard, the authors of [19] and [20], state that CNTs have the ability to move through tissues by forming pores via the apoplastic pathway and via endocytosis until reaching mesophyll cells in leaves, especially within chloroplasts, affecting photosynthetic activity by capturing carbon for energy use and electron transport. Ghasempour et al. [21], working with rose periwinkle (*Catharanthus roseu*) in vitro, at a concentration of 50 mg L^−1^ MWCNT, observed the highest amount of biomass, whereas at concentrations of 100 and 150 mg L^−1^ MWCNT they observed an increase in Chl content. Rahmani et al. [22], working with lilac sage (*Salvia verticillata*), observed an increase in Chl content at a concentration of 50 mg L^−1^ MWCNT.

### 3.3. Macro- and Micronutrient Content

The MWCNTs had an effect on the content of some macro- and micronutrients in sugarcane shoots cultured in vitro. As MWCNT concentrations increased, there was a tendency to increase the number of shoots per explant and the length and number of leaves per shoot. This increase in development could be associated with a greater absorption of N, Ca, S, Fe, and Zn; however, the low content of N, Fe, and Zn at a concentration of 200 mg L^−1^ MWCNT could be explained by different hypotheses: (1) a possible depletion of these elements in the culture medium that occurred during the early stages of shoot development, (2) due to greater diffusion caused by a dilution factor of these elements produced by a greater length and number of leaves per shoot and/or (3) due to an exchange of ions through the MWCNTs caused by a concentration gradient inside and outside the cell. This fact could also explain the low chlorophyll content obtained with the 200 mg L^−1^ MWCNT concentration, probably associated with a low amount of N and Fe, essential elements in chlorophyll synthesis. Similar results were reported by the authors of [23] in rice (*Oryza sativa* L.), where rapid root growth caused a significant decrease in N content, indicating that MWCNTs significantly decreased N assimilation. Another study [24] reported that K content decreased and Ca content increased during in vitro germination of maize (*Zea mays*) from 5 mg L^−1^ MWCNT. The Ca decreased from 20 mg L^−1^ MWCNT, whereas the content of P, Mg, and Na increased from 20 mg L^−1^ MWCNT and decreased from 50 mg L^−1^ MWCNT. The Fe content increased from 50 mg L^−1^ MWCNT and decreased from 200 mg L^−1^ MWCNT; S was not affected. These authors suggest that the increase and decrease of elements may be due to an ion exchange mechanism in the cell wall caused by MWCNTs. Taha et al. [25], found that on in vitro-grown date palm (*Phoenix dactylifera*) shoots, the content of N and P increased at the concentration of 0.1 mg L^−1^ MWCNT, whereas K and Ca had an increase at the concentration of 0.05 mg L^−1^ MWCNT; on the other hand, Na content decreased as the concentration of carbon nanotubes increased; this could demonstrate the ability of nanotubes to transport Na ions from the inside to the outside of cells.

The CNTs have the ability to transport heavy metals (Cu, Cd, Zn, Pb) without energy expenditure; however, the uptake of these metals depends on the CNT concentration, heavy metal, and plant genotype [12,19], simulating a chelating effect. Martinez-Ballesta et al. [10], working with broccoli (*Brassica oleracea* L.) protoplasts, observed that P and Fe content increased at 10 mg L^−1^ MWCNT.

This study demonstrated the potential use of carbon nanotubes during in vitro propagation of sugarcane, as they play an important role in plant development. Lahiani et al. [26] state that carbon nanotubes have the ability to penetrate plant cells and at high concentrations can act as a stress factor, affecting the overexpression of aquaporins, which are important transmembrane channels for transporting water and small solutes such as urea, ammonia, metalloids, gases, and even ions. Aquaporins are regulated in response to multiple hormonal and environmental stimuli, which contribute to various plant growth and development processes [27,28]. On the other hand, according to the authors of [29], the internalization of MWCNTs in cells could be through the pores of the cell wall, causing their expansion with different effects on plant development according to their concentration. Zhai et al. [30], working with soybean (*Glycine max*), demonstrated the presence of MWCNTs in the cytoplasm, cell wall, cell membrane, chloroplast, and mitochondria. According to the authors of [31], in wild sugarcane (*Saccharum spontaneum*) genotypes the pore diameter in cells ranges from 3.65 to 9.71 nm. The MWCNTs used in this study ranged in diameter from 6 to 13 nm. This indicates that a percentage of MWCNTs could probably penetrate and expand inside *Saccharum* spp. cells through the pores causing the effects described in this study. Furthermore, the results obtained suggest a hormetic effect, characterized by stimulation at low doses and inhibition at high doses; however, it is necessary to evaluate concentrations higher than 200 mg L^−1^ MWCNT. The hormetic effect in plants is related to the activation of antioxidant enzymes in response to a low production of reactive oxygen species (ROS) caused by a stressful stimulus. Another study [32], in tomato (*Solanum lycopersicum*), demonstrated an increase in antioxidant capacity at concentrations of 10–100 mg L^−1^ MWCNT and toxicity at 250–1000 mg L^−1^ MWCNT.

On the other hand, other authors state that MWCNTs could act as growth regulators, since at low concentrations they impact plant development [24,33]. In addition, the authors of [34] state that the effect of CNTs could be related to plant species, developmental stage, and growth conditions, as well as the type of nanotubes, their size and concentration.

This study showed that MWCNTs have an effect on shoot growth and differentiation, photosynthetic pigment content, and macro- and micronutrient content. In addition, MWCNTs have an effect on nutrient uptake, resulting in an increase in the multiplication rate of sugarcane shoots. These results open up the possibility of applying MWCNTs for in vitro multiplication in other crops of agricultural interest.

## 4. Materials and Methods

### 4.1. Characterization of Multi-Walled Carbon Nanotubes

Multi-walled carbon nanotubes (MWCNTs) (CAS: 308068-56-6) (Sigma-Aldrich^®^ Chemical Company, St. Louis, MO, USA) were used. The nanotubes (1 mg MWCNT) were dissolved in 1 mL isopropanol (Sigma-Aldrich^®^) and dispersed in two 10-min agitation cycles (Super Mixer, LAB LINE; Melrose Park, IL, USA) and 2 sonication cycles (Ultrasonic equipment, PS-20A, Shenzhen Jie Tai Co., LTD, Shenzhen, China) at 40 KHz, for 10 min at 25 °C. Morphological characterization of the nanotubes was performed under a transmission electron microscope (TEM) (Morgagni M-268, Philips/FEI, EI, NL). The MWCNT sample was taken in a 5 µL aliquot (1 mg MWCNT/10 mL sterile destined water) and deposited on a 200 mesh Formvar-carbon coated copper grid. Sample drying was performed at room temperature for 5 min. Microscope operating conditions were 80 Kv high voltage (HV), low magnification at 14,000X and high magnification at 200,000X, and column working pressure of 5 × 10^−3^ Pa (5 × 10^−5^ Torr). All micrographs were captured in .tiff format with a size of 1376 × 1032 pixels in grayscale format.

### 4.2. Establishment of In Vitro Sugarcane Explants

For the establishment of in vitro cultures, apices of approximately 30 cm in size were collected from four-month-old plants. The apices were reduced to a length of 15 cm and placed in thermo-hydrotherapy in a circulating thermostatic bath (Ecoshel, SC-15, McAllen, TX, USA) at 50 °C for 20 min. Subsequently, the apices were reduced to 1.5 cm, rinsed for five min in a 10% (*v*/*v*) solution of a commercial chlorine bleach, Cloralex™ (Industrias Alen, S.A. de C.V., Santa Catarina, NL, Mexico) (5% a.i.), with three drops of Tween 20^®^ (Sigma-Aldrich^®^) per 100 mL of water. The apices were rinsed three times with sterile distilled water. Finally, the explants were individually placed in test tubes containing 10 mL MS [35] culture medium supplemented with 30 g L^−1^ sucrose and without growth regulators. The pH of the culture medium was adjusted to 5.8; 0.25% (*w*/*v*) Phytagel™ (Sigma-Aldrich^®^) was added as a gelling agent and sterilized in the autoclave for 15 min at 120 °C and 115 kPa. The explants were incubated at 24 ± 2 °C, under irradiance of 40 ± 5 μmol m^−2^ s^−1^ and a photoperiod of 16 h of light. After one week of culture, the apices were transferred for the multiplication phase to MS culture medium supplemented with 30 g L^−1^ sucrose, 1 mg L^−1^ Kinetin (KIN), 0.6 mg L^−1^ Indoleacetic acid (IAA) and 0.6 mg L^−1^ Benzylaminopurine (BAP). All reagents were Sigma-Aldrich^®^ products. After three subcultures (30 d each) in the multiplication phase in semisolid medium, shoots were taken as an explant source for the experiments with MWCNTs.

### 4.3. Application of Carbon Nanotubes in In Vitro Cultures

The semi-automated bioreactor used was a temporary immersion bioreactor (TIB) described by Escalona et al. [36]. The bioreactor consists of two jars; a jar for growing explants and the other jar containing the culture medium, the jars are connected with a platinum-cured silicone tubing. Air pressure is applied to the medium container to immerse the explants. After immersion time, a solenoid valve prevents the passage of air and the medium returns to the original jar. The TIBs with 1000 mL capacity were used. The jar of culture medium was filled with 500 mL of culture medium and sterilized at 120 °C for 20 min. In the jar of explants were placed ten explants (2 shoots each) with a length of 2 cm. The immersion time was 2 min every 8 h for 30 days. Incubation conditions were the same as described above.

### 4.4. Evaluation of Macro- and Micronutrient Content

To determine macro- and micronutrient content, samples were dried at 70 °C in a drying oven for 72 h and pulverized in a blender (Oster 6832, Milwaukee, WI, USA). Samples were subjected to wet digestion in a mixture of perchloric and nitric acids at a 2:1 (*v*/*v*) ratio, according to the protocol described by Alcántar and Sandoval [37]. To determine the concentrations of macronutrients (P, K, Ca, Mg, and S) and micronutrients (B, Cu, Fe, Mn, Ni, and Zn), the extracts were analyzed using a coupled plasma induction optical emission spectrometer (ICP-OES, Varian 725-ES, Agilent; Mulgrave, VIC, Australia). The N concentration was determined by the semi-microkjeldahl method according to the protocol described by Bremner [38].

### 4.5. Quantification of Carbon Content

The C content was determined according to the authors of [39] method. Samples of 100 mg from each treatment were subjected to digestion in a mixture of 10 mL of 1 N potassium dichromate and 20 mL of 0.1 N concentrated sulfuric acid. Subsequently, the mixture was carried out in 500 mL Erlenmeyer flasks and shaken manually for 1 min. The C quantification was performed by titration with 0.05 N ferrous sulfate heptahydrate.

### 4.6. Chlorophyll Content

Total chlorophyll content was determined according to the methodology proposed by Harborne [40]. For each sample, 1 g of fresh matter was macerated with 80% acetone and left to stand at −4 °C for 24 h in 80% acetone at final volume of 10 mL. Subsequently, the mixture was filtered with No. 41 filter paper and adjusted to a volume of 25 mL with 80% acetone. Two mL per sample were used at an absorbance of 663 and 645 nm for chlorophyll a and b, respectively. Readings were performed using a spectrophotometer (Genesys 10S, Thermo Scientific, Waltham, MA, USA). Finally, quantification was performed using the following Equation (1):

Total chlorophyll = [[(8.20 × A663)−(20.20 × A645)] (V)]/(1000 × W)(1)

where A645 and A645: absorbance, V: graduation volume in mL^−1^, W: sample weight in g, and 1000 is the conversion factor.

### 4.7. Experimental Design and Statistical Analysis

A completely randomized experimental design was used. All experiments were performed in triplicate. Data were subjected to one-way analysis of variance (ANOVA) and Tukey’s mean comparison (*p* ≤ 0.05) using IBM SPSS^®^ statistical software (version 22 for Windows).

## 5. Conclusions

In conclusion, it was demonstrated that the administration of MWCNTs at low concentrations produce favorable physiological effects on development in the in vitro multiplication stage of sugarcane using temporary immersion bioreactors. These results demonstrated that MWCNTs can induce a hormetic effect during in vitro shoot multiplication of sugarcane and may have a potential use in other species. In addition, MWCNTs provide applications to improve crop production by increasing the efficiency during micropropagation.

## Figures and Tables

**Table 1:** Effect of multi-walled carbon nanotubes on macro- and micronutrient content of in vitro shoots of sugarcane (*Saccharum* spp.) cv. Mex 69-290 in temporary immersion.

**MWCNT (mg L** **^−1^)**	**Macronutrients (mg kg** **^−1^)**						
	**N**	**P**	**K**	**Ca**	**Mg**	**S**	
0	47,900 ± 1300 ^a^	3288.13 ± 63.92 ^a^	39,156.96 ± 3609.36 ^a^	1425.42 ± 25.07 ^b^	1006.97 ± 20.32 ^a^	3032.37 ± 72.02 ^b^	
50	45,800 ± 1400 ^a^	3172.63 ± 84.08 ^a^	39,263.50 ± 4270.20 ^a^	1561.81 ± 14.68 ^b^	1008.41 ± 16.54 ^a^	3267.52 ± 13.44 ^ab^	
100	42,400 ± 1500 ^ab^	3170.66 ± 126.87 ^a^	36,006.56 ± 3114.44 ^a^	1627.85 ± 60.79 ^ab^	1006.35 ± 8.31 ^a^	3549.03 ± 69.46 ^a^	
200	39,500 ± 1600 ^b^	3304.98 ± 108.87 ^a^	36,434.70 ± 4068.33 ^a^	1848.00 ± 70.56 ^a^	1051.42 ± 27.62 ^a^	3487.07 ± 122.15 ^a^	
	**Micronutrients (mg kg** **^−1^)**						
	**Fe**	**Cu**	**Zn**	**Mn**	**B**	**Ni**	**Na**
0	299.49 ± 7.04 ^ab^	6.41 ± 0.38 ^a^	69.73 ± 0.25 ^b^	62.04 ± 0.86 ^a^	20.75 ± 0.43 ^a^	1.42 ± 0.26 ^a^	508.29 ± 10.86 ^a^
50	318.00 ± 3.79 ^a^	3.32 ± 0.35 ^b^	74.45 ± 0.93 ^ab^	62.89 ± 0.64 ^a^	20.17 ± 0.64 ^a^	2.11 ± 0.50 ^a^	515.24 ± 9.16 ^a^
100	322.49 ± 2.45 ^a^	3.22 ± 0.17 ^b^	76.65 ± 0.44 ^a^	61.39 ± 0.42 ^a^	20.20 ± 0.29 ^a^	1.58 ± 0.90 ^a^	322.45 ± 1.50 ^b^
200	281.86 ± 10.04 ^b^	3.39 ± 0.02 ^b^	68.98 ± 2.61 ^b^	64.65 ± 2.61 ^a^	22.45 ± 0.93 ^a^	2.51 ± 0.79 ^a^	352.95 ± 16.30 ^b^

Results are shown as mean ± SE (standard error). Means with a different letter are significantly different (Tukey, *p* ≤ 0.05).

## Data Availability

Not applicable.
